# Therapeutic implications of cancer stem cells in prostate cancer

**DOI:** 10.20892/j.issn.2095-3941.2022.0714

**Published:** 2023-06-05

**Authors:** Pinaki Banerjee, Prachi Kapse, Shehnaz Siddique, Moumita Kundu, Jasoda Choudhari, Varshasnata Mohanty, Diksha Malhotra, Suresh W. Gosavi, Rajesh N. Gacche, Gopal C. Kundu

**Affiliations:** 1 National Centre for Cell Science, Savitribai Phule Pune University Campus, Pune 411007, India; 2 School of Basic Medical Science, Savitribai Phule Pune University, Pune 411007, India; 3 School of Biotechnology, KIIT Deemed to be University, Bhubaneswar 751024, India; 4 Department of Biotechnology, Savitribai Phule Pune University, Pune 411007, India; 5 Kalinga Institute of Medical Sciences (KIMS), KIIT Deemed to be University, Bhubaneswar 751024, India

**Keywords:** Epithelial-mesenchymal transition, metastasis, prostate cancer, cancer stem cells, tumor growth, tumor microenvironment, signaling

## Abstract

Prostate cancer, one of the most frequently occurring cancers in men, is a heterogeneous disease involving multiple cell types within tumors. This tumor heterogeneity at least partly results from genomic instability leading to sub-clonal cellular differentiation. The differentiated cell populations originate from a small subset of cells with tumor-initiating and stem-like properties. These cells, termed prostate cancer stem cells (PCSCs), play crucial roles in disease progression, drug resistance, and relapse. This review discusses the origin, hierarchy, and plasticity of PCSCs; methods for isolation and enrichment of PCSCs; and various cellular and metabolic signaling pathways involved in PCSC induction and maintenance, as well as therapeutic targeting.

## Introduction

Prostate cancer has the second-highest incidence rate and fifth-highest mortality rate among cancers affecting the world’s male population^1,2^. Despite multiple advances in early detection and the availability of numerous prostate cancer treatments, limited progress has been made in treating locally advanced and metastatic forms of the disease. Most manifestations of prostate cancer initially respond to androgen deprivation therapy (ADT); however, many cases develop into an androgen-refractory form of the disease^3^. The heterogeneous population of cells within prostate tumors explains the many uncertainties involving the etiology and treatment of advanced disease.

Hanahan and Weinberg^4^, in 2000, described 6 hallmarks of cancer: insensitivity to anti-growth signals, evasion of apoptosis, limitless replicative potential, self-sufficiency in growth signals, sustained angiogenesis, and tissue invasion and metastasis. In 2011, they added another 4 emerging hallmarks: evasion of immune destruction, reprogramming of energy metabolism, tumor-promoting inflammation, and genomic instability and mutation^5^. However, with new knowledge and a better understanding of multiple aspects of tumorigenesis and disease progression, the hallmarks of cancer have been redefined as selective growth and proliferative advantage; altered stress responses favoring overall survival, vascularization, invasion, and metastasis; metabolic rewiring; a favorable microenvironment; and immune system modulation^6^. Prostate cancer stem cells (PCSCs), a cell population with self-renewal properties, eventually accumulate multiple heterogeneous mutations over time and consequently display several hallmarks of cancer, including resistance to various treatments^7^. The cancer stem cell (CSC) model of prostate cancer has gained attention in recent years because of its relevance to cancer prognostication and treatment^8^. In its basic form, the CSC model is hierarchical: CSCs reside at the top and possess the unlimited self-renewal ability, and progeny cells become increasingly differentiated and lose their tumorigenic properties in the process^9^. In contrast, the stochastic concept of tumor heterogeneity proposes that all cells within a tumor have high tumorigenicity and bear various mutations and epigenetic modifications. These distinct concepts are not mutually exclusive and can coexist under an umbrella model involving multiple lineages of CSCs within a tumor, which undergo clonal evolution^10^.

The various signaling pathways that maintain homeostasis in healthy stem cells are often deregulated in cancer. The affected pathways associated with AKT, MAPK, Hedgehog, Notch, WNT, and Hippo, contribute to the formation and maintenance of CSCs in prostate cancer. Metabolic pathways and their reprogramming have been reported to participate in crosstalk with various molecular pathways and downstream transcription factors crucial for the induction and maintenance of CSCs. The metabolites, through multiple interactions, also modify the cellular niche, thus producing a CSC-favorable environment^11^. The Warburg effect allows cancer cells to utilize non-mitochondrial energy and essential by-product-producing pathways, through an aerobic glycolysis mechanism. The downregulation of oxidative phosphorylation (OXPHOS) pathways is associated with epithelial-to-mesenchymal transition (EMT) and promotes the conversion to a CSC phenotype^12^.

The therapeutic targeting of PCSCs in prostate cancer management is complex. These PCSCs usually lack androgen receptors (ARs) and thus do not respond to androgen hormone depletion therapy. Owing to inherent genetic instability, tumors can evolve new variants that become responsible for hormone-refractory disease^7^. The various signaling pathways involved in PCSC maintenance, in conjunction with the activity of ATP-binding cassette (ABC) transporters, have been implicated in conferring drug resistance on PCSCs. Cancer immunotherapy has achieved remarkable successes in recent years, and the cell surface markers that identify PCSCs have become targets for immunotherapies^13^. However, PCSCs acquire multiple mutations, thus enabling their evasion of immune surveillance and immunotherapy. Such mutations also confer adaptability and metastatic potential. Some commonly arising mutations include E26 transformation-specific (ETS) fusions, deletion of the NKX3-1 gene, and increased copy numbers of MYC and other genes^14^. This review focuses on understanding PCSCs by studying their origin, models, and various metabolic and signaling crosstalk associated with stem cell induction and maintenance and on describing recent therapeutic approaches to target these cells.

## PCSCs: origin, hierarchy, heterogeneity, and plasticity

Normal prostate tissue comprises 3 types of cells: luminal, basal, and neuroendocrine^15^. Mutations in these cell types can lead to uncontrolled cell division, which then develops into prostate cancer. According to literature reports, tumor initiators usually originate from the prostate epithelium’s basal cell layer or luminal cells. Recent studies by Zhang et al.^16^ have revealed the contribution of luminal progenitor cells to prostate cancer development and their role as drivers of tumor progression. A subset of cells known as CSCs exists within tumors and exhibit features of longevity, multipotency, and self-renewal^17^. CSCs also possess proliferative and regenerative capabilities and the potential for initiation of diversification and drug resistance^18^.

A distinct stem-cell-like population within prostate cancer has the tumor-initiating ability and conveys castration resistance^19^. Basal prostate stem cells express cell surface markers, including cytokeratin 5, cytokeratin 14, p63, integrin α2β1, the cluster of differentiation 133 (CD133), and the cluster of differentiation 44 (CD44). They express less AR as compared to metastasized prostate cancer cells. This explains the CSC hierarchy^20–22^. Healthy prostate tissue stem cells have been postulated to arise from the basal compartment. In murine models, the basal population preferentially survives under androgen-depleted conditions, whereas most luminal-origin cells undergo apoptosis^23,24^. These normal prostate stem cells give rise to progenitor cells, and differentiated cells eventually arise through favoring of asymmetric cell division.

**Figure 1:**
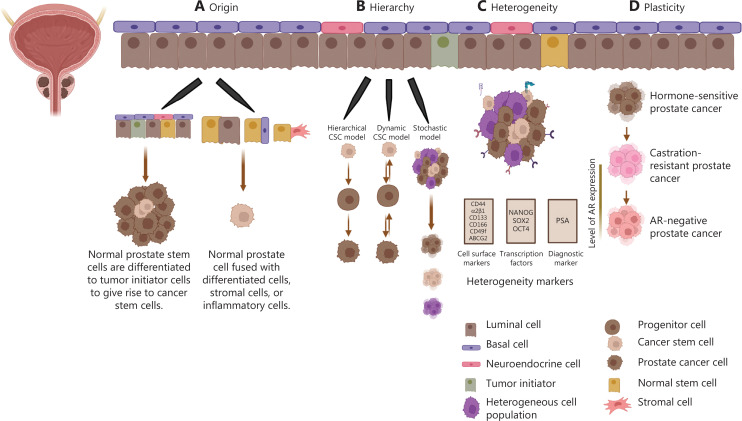
PCSC origin, hierarchy, heterogeneity, and plasticity. (A) Schematic of the prostate gland, showing prostate cancer stem cells, progenitor cells, basal cells, luminal cells, and neuroendocrine cells. The fusion of differentiated, stromal, and inflammatory cells with normal prostate cells leads to the generation of cancer stem cells. Recent studies have indicated that luminal cells are the cells of origin. (B) Various hierarchical models, such as the dynamic CSC model, stochastic model, and hierarchical stem cell model, explain hypotheses regarding the origin of prostate cancer. (C) Heterogeneity among tumors results in the presence of diverse cell populations expressing various heterogeneity markers such as CD44, α2β1, CD133, CD166, CD49f, ABCG2, NANOG, PSA, SOX2, and OCT4. (D) Plasticity explains the conversion of hormone-sensitive prostate cancer into castration-resistant prostate cancer, thus ultimately generating AR-negative prostate cancer.

In contrast, both the basal and luminal cell populations can be cells of origin of PCSCs^25^. In human prostate cancer, basal cells have been reported to be the most likely origin^26^. Multiple crucial molecules such as p63, Bcl-2, and hTERT have been well documented to preferentially localize in the basal cell population of the prostate, thus giving rise to an alternative hypothesis in which PCSCs arise from normal prostate stem cells that have undergone malignant transformation. This possibility is supported by observations in basal cells in most metastatic prostate cancers^27–29^. However, luminal cells have also been postulated to be a potential origin of prostate cancer. Castration-resistant Nkx3.1-expressing cells, which are of luminal origin, have been demonstrated to be the cell of origin in some types of prostate cancer^30^. Researchers have also reported that human prostate cancer is primarily luminal and have described the role of prostate luminal progenitor cells in tumorigenesis. However, the link between luminal cells and the PCSC cell of origin remains unclear because studies have been limited by challenges in stem cell-associated bioassays^16^. Another proposed origin of PCSCs is that they might arise from a fusion between normal prostate stem cells and other cancerous cells, including differentiated cells, stromal cells, or inflammatory cells. This hypothesis might explain why PCSCs have the self-renewal ability while also bearing the accumulated mutations present in differentiated cells, thus completing neoplastic transformation^31^ (**Figure 1A**). De novo or ADT-dependent occurrence of neuroendocrine prostate cancer (NEPC) often generates more stem-like cells. In NEPC, genomic and epigenetic changes lead to the upregulation of stemness genes, such as SOX2 and c-Myc, and the downregulation of prostate-specific antigen (PSA) and AR expression. Epigenetics is also crucial in promoting prostate cancer development and metastasis^32^. EZH2-mediated EMT leads to the enhancement of stemness properties and the progression of NEPC^33^.

The hierarchical stem cell model describes the unidirectional conversion of CSCs into progenitor cells, thereby giving rise to non-CSCs and multipotent cells. Dynamic CSC models can be characterized by the conversion of non-CSCs into CSCs and vice versa. In both hierarchical and dynamic models, CSCs have tumor-initiating potential (**Figure 1B**). Chromatin remodeling and histone modifiers control a dynamic equilibrium between CSCs and non-CSCs^34^. Castration-resistant prostate cancer (CRPC) has a phenotype of aldehyde dehydrogenase^+^ (ALDH^+^), CD44^+^, and α2β1^+/hi^ ^35^. Patrawala et al.^36^ have reported that CD44^+^ enriched tumor cells develop xenografts more rapidly than CD44^-^ cells, and α2β1^+/hi^ cells show higher clonogenicity than α2β1^-/lo^ cells *in vitro*. Analysis of patient samples has indicated that 70% of the cell population expresses both CD44 and α2β1 cell surface markers.

A tumor may have a single point of initiation due to mutations within normal stem cells, thus resulting in loss of control over the self-renewable phenotype^37^. CSCs and circulating tumor cells express stemness factors, such as TWIST, SLUG, and SNAIL, which promote aggressiveness and metastasis^38^. Prostate cancer sub-types of basal cell origin give rise to squamous carcinoma and adenocarcinoma. Myristoylated AKT1 (myr-AKT1) overexpression and N-MYC mutation result in the development of prostate adenocarcinoma, squamous carcinoma, and NEPC. Conditional deactivation of CK14-creER, P53, Smad4, and PTEN tumor suppressor genes in basal cells, along with ARR2PB-Cre and CK8-CreER in luminal cells, gives rise to the development of prostate adenocarcinoma. Inactivation of the RB1, PTEN, and P53 genes leads to NEPC growth. Mice bearing PTEN/RB1-deficient prostate adenocarcinoma, after undergoing castration or abiraterone treatment of PTEN/P53-deficient prostate adenocarcinoma, show generation of NEPC^14^. Non-treated prostate tumors express both AR and PSA. PSA, a biomarker used primarily for prostate cancer diagnosis, is a downstream target of AR^39^. After clonal evolution, with ADT, the expression of PSA decreases, and some cells undergo apoptosis, thus indicating tumor regression. High-grade prostate tumors exhibit low PSA expression, and AR therapy-resistant cells survive and develop castration-resistant cancer^40^. CRPCs developed after ADT have been found to express NK3 homeobox 1 (NKX3-1). These cells exhibit expression of other stem cell-like markers, such as CK18, and may serve as cells of origin^30^. Beyond PSA, CD44, CD49f, and CD49b can also be used as lineage-specific markers to identify the origin of PCSCs^41^ (**Figure 1C**). Various paracrine growth factors, such as secreted frizzled-related protein 1 (SFRP1), stromal cell-derived factor 1 (SDF-1), and transforming growth factor-β1 (TGFβ1), reside within the tumor-stroma microenvironment and promote the invasion of prostate cancer^42–44^. These factors have been found to activate anti-apoptotic pathways and promote invasion. PCSC phenotypes are also regulated by downstream targeting of the NFκB and JAK-STAT pathways by these factors^45^.

Stable integration of the hTERT vector into the human prostate epithelial (HPE) cell line led to the establishment of the HPET cell line (where T indicates hTERT). A study using HPE cell lines has provided an understanding of the development of prostate cancer after AR deprivation. The HPET cell line unexpectedly has been found to express stemness factors, such as SOX2, NANOG, OCT4, Nestin, CD44, and CD133, but not p63 and AR. HPET retains the expression of all 3 types of epithelial cells and develops into prostate tumors^25^. Although the mechanism driving lineage plasticity in prostate cancer remains unclear, plasticity within the epithelial cell population of a mixed basal-luminal phenotype depends on JAK-STAT and FGFR signaling. Single-cell analysis has confirmed that JAK/STAT and FGFR signaling determines lineage plasticity in prostate cancer. Keratin 13 (KRT 13) is enriched in prostate stem cells at single-cell resolution, according to prostasphere-based label retention assays. Single-cell RNA-seq analysis has revealed 3 clusters of PCSCs: cluster I (PSCA, CD36, SPINK1, and KRT13/23/80/78/4 enriched) representing quiescent stem cells; cluster II representing active stem cells; and cluster III (KRT16/17/6 enriched) representing bipotent progenitor cells^44^.

Luminal progenitors serve primarily as tumor initiators or cells of origin for prostate cancer. Various hierarchical models have been proposed. A recent model suggests that, during early postnatal development, multipotent basal cells (p63^+^ CK5^+^) differentiate into unipotent basal progenitors, luminal progenitors (CK8^+^ AR low), and NE cells. These unipotent basal and luminal progenitors can undergo self-renewal or differentiate into basal cells and mature luminal cells (AR^+^ CK8^+^), respectively. In adults, during homeostasis and regeneration, the bipotent basal progenitor forms unipotent basal and luminal progenitors, which further undergo self-renewal. Unipotent basal progenitors differentiate into basal cells, and luminal cell progenitors differentiate into luminal cells.

In contrast, different groups have reported that bipotent basal progenitors might also give rise to basal and luminal cells. Tumor heterogeneity at various levels—such as the epigenetic, post-translational, morphological, and phenotypic levels—can be examined to assess clonal and subclonal changes^45^. Heterogeneity at the genetic level poses diagnostic challenges, and sequencing studies have indicated that individual tumor foci can give rise to clonally distinct lesions without sharing driver gene alterations. Acquired drug resistance in prostate cancer changes the cell phenotype, and AR-independent pathways are adopted for growth and survival (**Figure 1D**). The aggressive prostate tumor results in epithelial to mesenchymal plasticity during reactivation of the developmental program. We have established an understanding of the link between NEPC emergence and plasticity. Enhanced knowledge of prostate cancer is expected to lead to improvements in treatment and clinical management^46^.

## Current methods for PCSC enrichment and analysis

**Figure 2:**
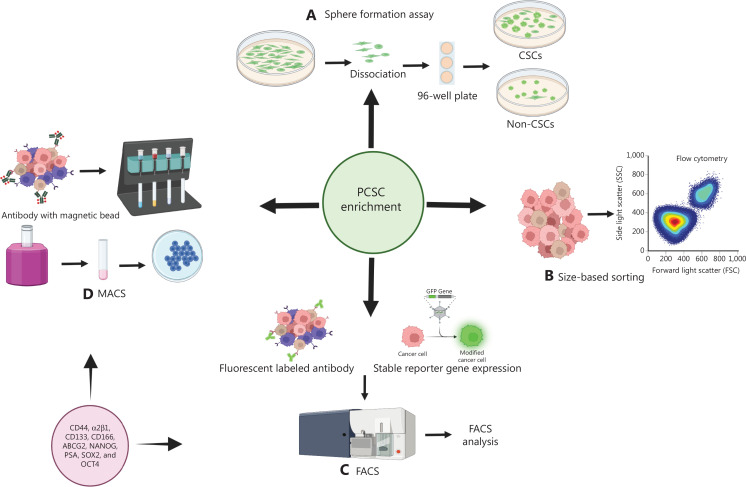
Enrichment of PCSCs. (A) Sphere formation assays: CSCs can form spheres. (B) Size-based cell sorting, based on the smaller size of CSCs than cancer cells. (C) Fluorescence-associated cell sorting (FACS), used for sorting PCSCs by using fluorescently labeled antibodies. (D) Magnetic-activated cell sorting (MACS), in which antibodies that bind PCSC markers are labeled with magnetic beads.

PCSCs express specific markers that can be used to isolate and enrich these cell populations. Enrichment can be achieved by sorting cells from prostate cancer cell lines and patient tissues by identifying specific surface markers. Prostate stem/progenitor cells have been reported to express various cell surface markers such as CD44, integrin α2β1, CD133, CD166, and ATP-binding cassette sub-family G member 2 (ABCG2)^47^ (**Table 1, Figure 2**). Cells expressing CD44^+^ along with α2β1^+/hi^ are as tumorigenic as CD44^+^ and α2β1^-/lo^ cells. CD44^-^ and α2β1^+/hi^ cells exhibit more tumorigenic potential than CD44^-^ and α2β1^-/lo^ cells. CD44 is a prominent stemness marker, whereas α2β1 is more aligned with the hierarchical depiction of the PCSCs and their metastatic characteristics^36^. “Side populations” obtained through Hoechst dye exclusion assay represent stem cell phenotypes. Isolation and enrichment of CSCs can be performed with Hoechst 33342 and Rhodamine 123^48^. Strategically repeated chemotherapy and radiotherapy increase the numbers of cancer cell-rich in CSC markers, mainly because of acquired treatment resistance. Enriched chemotherapy-resistant carcinoma cells are tumorigenic, metastatic, and highly aggressive. A particular treatment-resistant CSC population is enriched in the ABC transporter protein ABCG2. Tumor cells from cell lines and patient-derived xenografts can be analyzed for ABCG2 enrichment through flow cytometry sorting of cancer cells with high efflux of dyes such as Hoechst 33342 and Rhodamine 123^42,43^. Studies have suggested that CSCs may be identified by selecting a marker-based population or de-differentiation of cells.

**Table 1:** Various markers for prostate cancer stem cells and methods of enrichment/analysis

Stemness marker	Method of enrichment/analysis	Effect	Stemness marker in other cancer	Ref.
**CD44**	FACS, MACS, sphere formation assay, immunofluorescence microscopy, IHC	Tumor progression Self-renewal ability Expression of stemness genes Metastasis	Colorectal cancer, lung cancer, breast cancer, leukemia, pancreatic cancer, head and neck cancer	^180^ ^181^ ^182^ ^19^
**CD133**	FACS, MACS, sphere formation assay, immunofluorescence microscopy, IHC	Tumor progression Self-renewal ability Expression of stemness genes	Brain cancer, colorectal cancer, lung cancer, ovarian cancer, liver cancer	^180^ ^181^ ^182^
**CD117/c-kit**	FACS, MACS, sphere formation assay, immunofluorescence microscopy, IHC	Tumor progression Metastasis Recurrence and therapeutic resistance	Gastrointestinal stromal cancer, melanoma, small cell lung carcinoma, leukemia	^183^ ^184^ ^183^
***α*_2_*β*_1_ integrin**	FACS, MACS, sphere formation assay, immunofluorescence microscopy, IHC	Tumor progression Self-renewal ability Recurrence and therapeutic resistance	Colorectal cancer, non-small cell lung cancer	^185^ ^186^ ^185^
***α*_6_ integrin**	FACS, MACS, sphere formation assay, immunofluorescence microscopy, IHC	Tumor progression Self-renewal ability Recurrence and therapeutic resistance	Glioblastoma	^185^ ^181^ ^185^
**CXCR4**	FACS, sphere formation assay, immunofluorescence microscopy, IHC	Tumor progression Self-renewal ability Recurrence and therapeutic resistance Metastasis	Leukemia, brain cancer, breast cancer, retinoblastoma, ovarian cancer, cervical cancer	^187^ ^188^ ^189^
**CD166**	FACS, MACS, sphere formation assay, immunofluorescence microscopy, IHC	Tumor progression Self-renewal ability Recurrence and therapeutic resistance	Bladder cancer, breast cancer, colorectal cancer, lung cancer, head and neck cancer, ovarian cancer, melanoma	^190^ ^190^ ^190^
**ABCG2**	Immunoblotting, qPCR	Recurrence and therapeutic resistance	Pancreatic cancer, melanoma, glioma	^191^
**ALDH1**	FACS	Tumor progression Self-renewal ability Recurrence and therapeutic resistance Expression of stemness genes	Liver cancer, lung cancer, breast cancer, colorectal cancer, pancreatic cancer, ovarian cancer	^192^ ^192^ ^193^ ^193^
**EZH2**	Immunoblotting, qPCR	Tumor progression Metastasis Recurrence and therapeutic resistance Expression of stemness genes	Breast cancer, pancreatic cancer, ovarian cancer, melanoma, colorectal cancer, leukemia, hepatocarcinoma	^194^ ^195^ ^196^ ^194^
**Trop2**	FACS, MACS, sphere formation assay, immunofluorescence microscopy, IHC	Tumor progression Self-renewal ability	Pancreatic cancer, ovarian cancer, lung cancer, breast cancer, colorectal cancer	^197^ ^198^
**PSA**	Gene reporter system-based FACS, immunofluorescence microscopy	Tumor progression Self-renewal ability Recurrence and therapeutic resistance Expression of stemness genes	-	^35^ ^35^ ^35^ ^199^

CSCs within the side population are highly aggressive and possess tumor initiation and self-renewal abilities, and hence can develop into heterogeneous tumors. Fluorescence-activated cell sorting (FACS) or magnetic cell sorting (MACS) techniques can be used to sort CSCs by using antibodies tagged with different fluorophores or magnetic beads for phenotypic separation, respectively^49,50^. The absence of specific cell surface markers, such as CD24, may also be used to sort CSCs across different cell lines, xenograft tumors, and patient-derived specimens^51^. Hence, marker-based studies of CSC populations are often reliable and specific. A detailed understanding of CSC characteristics is needed to improve the efficacy and accuracy of CSC-based studies.

Although stem cells may be characterized according to cell surface markers, they can also be sorted by reporter gene assays using the promoters of intracellular PCSC markers such as NANOG, PSA, SOX2, and OCT4^52–55^. PSA high or PSA low cells, or cells with no PSA, vary in their biological functionality. Clinical studies have revealed that prostate cancer cells with low PSA expression are resistant to various drugs and anti-androgen therapy. Evidence has suggested that a PSA low population mimics the characteristic features of PCSCs^55^.

Li et al.^56^ have reported that CSCs can be more invasive than cancer cells. Sphere formation is a cell adhesion-independent process. Cells forming spheres for multiple passages are self-renewing and highly proliferative, showing spherogenicity for numerous generations. Sphere-forming cells have been found to have intracellular and cell surface CSC-marker expression^57^.

## Signaling networks involved in CSC maintenance

**Figure 3:**
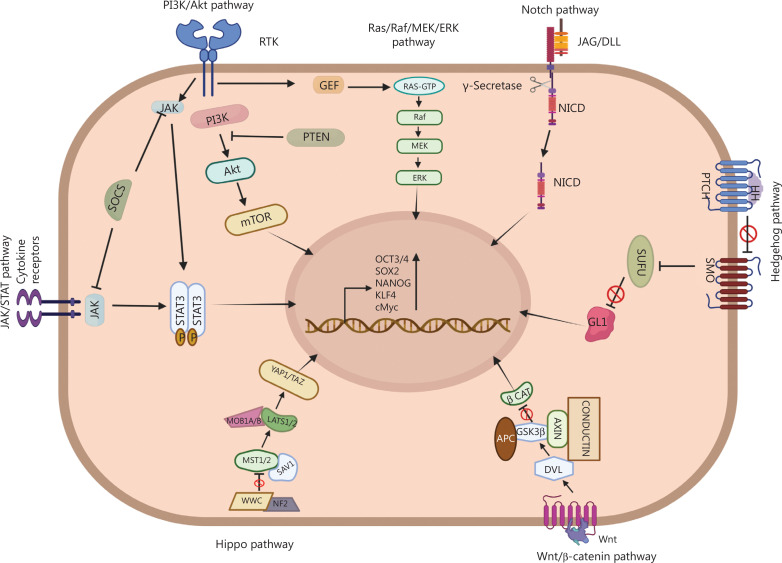
Various signaling pathways, such as JAK/STAT, WNT/β-catenin, Hippo, Notch, Hedgehog, PI3K/AKT, and Ras/Raf/MEK/ERK, both individually and through crosstalk, regulate the transcription of OCT3/4, SOX2, NANOG, KLF4, and cMyc, thus maintaining the stemness of the prostate cancer stem cells.

The molecular pathways involved in maintaining normal stem cell homeostasis are often deregulated in CSCs. Such abnormal signaling is involved in self-renewal, differentiation, proliferation, and drug resistance in CSCs. These pathways crosstalk with other signaling pathways involved in various extrinsic and intrinsic processes^58^. Several signaling cascades, such as Notch, WNT, Hedgehog, Hippo, PI3K/AKT, RAS/MAPK, and STAT3, have been reported to maintain PCSCs^59^ (**Figure 3**).

### PI3K/AKT signaling

PI3K, a frequently dysregulated signaling pathway in most cancers, increases EMT properties, drug resistance, and stemness. PI3K stimulates the mammalian target of rapamycin (mTOR) through the activation of AKT. PTEN, which negatively regulates PI3K/AKT signaling, is often mutated or deleted in multiple cancers^3^. Loss of PTEN and overexpression of PI3K/AKT induce stem-like properties and sphere formation in prostate cancer cells, such as LNCaP, DU145, and PC3. Elevated AKT activity has been observed in clinical specimens with a Gleeson grading of 8 and higher^59^. PTEN knockdown in prostate cancer cell lines increases sphere-forming properties and enrichment in CD44^+^/CD133^+^ cells^60^. Increased PI3K/AKT signaling pathway activity is also associated with resistance to radiotherapy by enhancing CSC and EMT phenotypes^61^. AKT1/2 has been reported to be involved primarily in regulating chemoresistance in prostate cancer^62^.

### RAS/MAPK signaling

MAPKs are evolutionarily conserved cytoplasmic serine/threonine kinases. The MAPK signaling pathway responds to extracellular stimuli, thereby regulating fundamental cellular processes such as cell growth, proliferation, migration, differentiation, and apoptosis^63^. MAPK signaling is responsible for stem cell characteristics in prostate cancer cells. DU145 cells exhibit a decrease in sphere-forming ability after treatment with the MEK inhibitor U0126 or knockdown of ERK^64^. RAS activation, PI3K/AKT activation, and loss of PTEN induce an increase in the EMT phenotype and macro-metastasis in prostate cancer^65^. Aberrant fibroblast growth factor receptor (FGFR) signaling has been reported to induce EMT and stemness in prostate cancer cell lines by activating the RAS/MAPK, PI3K/AKT, and JAK/STAT pathways. High expression of p-AKT, p-STAT5, and p-MAPK has also been reported in spheroids generated from PC3, DU145, and LNCaP cells^66^.

### Signal transducer and activator of transcription 3 (STAT3) signaling

Upregulated STAT signaling has been well documented in prostate cancer, and elevated STAT3 activation has been observed in prostate cancers and adjacent normal prostate tissues^67^. However, STAT3 signaling has been recently reported to be responsible for the CSC phenotype in prostate cancer cells. Loss of AR results in IL6-mediated STAT3 activation. Activated STAT3 induces the development of PCSCs^68^. α2β1^hi^/CD133^+^ cells isolated from human prostate cancer patient samples also show elevated expression of IL6 and STAT3^69^.

### Notch signaling

Notch signaling regulates differentiation in the benign prostate and determines the structure of the prostate gland. However, in prostate cancer, activated Notch signaling increases the survival of cancer cells. The expression of Notch pathway-associated proteins such as Jagged2, Notch3, and Hes6 is enhanced in higher grades of cancer^70^. Deregulated Notch signaling in prostate cancer is responsible for tumor recurrence, resistance to treatment, EMT phenotype, and stem-like properties^71^.

### Hedgehog signaling

Aberrant Hedgehog signaling has been associated with the development of different types of cancers and implicated in many aspects of tumorigenesis, including CSC maintenance^72^. During prostate development, autocrine and paracrine Hedgehog signaling regulates growth and differentiation. Hedgehog signaling is responsible for the enrichment of stemness through autocrine and paracrine mechanisms in prostate cancer^73^. Hedgehog activity increases with higher prostate cancer grades and is responsible for EMT and subsequent metastasis^74^. Aberrant Hedgehog signaling induces drug resistance in CD133^high^/CD44^high^ PCSCs^75^.

### WNT signaling

Dysregulation or mutation of various genes associated with the WNT pathway has also been associated with cancer development and progression^76^. The WNT pathway has been extensively associated with the self-renewal ability of prostate cancer cells and other stem-like characteristics. Inhibition of the WNT pathway decreases both sphere size and self-renewal ability in prostate cancer cell lines. Alternatively, an increase in WNT3a has been associated with the increased sphere-forming ability of prostate cancer cells^77^. WNT/β-Catenin signaling induces self-renewal and symmetric cell division in hTERT^high^ prostate cancer cell lines. hTERT^high^ cells exhibit CSC characteristics, such as sphere formation and elevated expression of CSC markers^78^. Various CSC markers are often directly regulated by the WNT/β-Catenin signaling axis. Dysregulated WNT signaling enhances CD44 and ALDH1A expression at the mRNA and protein levels^79^. miR-605, a microRNA inhibiting WNT signaling through Keratin 5 (KRT5), decreases the proliferation, migration, and invasion of PCSCs^80^.

### Hippo signaling

The Hippo signaling axis consists of highly conserved kinases acting in a cascade (MST1/2 and LATS1/2) and the downstream effector proteins YAP and TAZ. This signaling pathway is crucial for cellular homeostasis and tissue regeneration by stem cell regulation. Dysregulation of this pathway is intrinsically associated with tumor development, growth, and progression by CSC enrichment^81^. ANKHD1, a positive regulator of YAP1, is overexpressed in prostate cancer cells. Silencing of ANKHD1 leads to the downregulation of YAP1 and decreases prostate cancer cell growth and progression^82^. YAP1 also regulates the self-renewal property of prostate cancer cells and is negatively regulated by AR *via* YAP1 promoter methylation. ADT leads to the loss of function of AR, thus activating YAP1 and inducing CSC-like characteristics^83^. Silencing of TAZ, another core downstream effector of the Hippo pathway, decreases colony formation ability, CSC expression, and overall stemness in PCSCs^84^.

## Interaction between PCSCs and the tumor microenvironment (TME)

**Figure 4:**
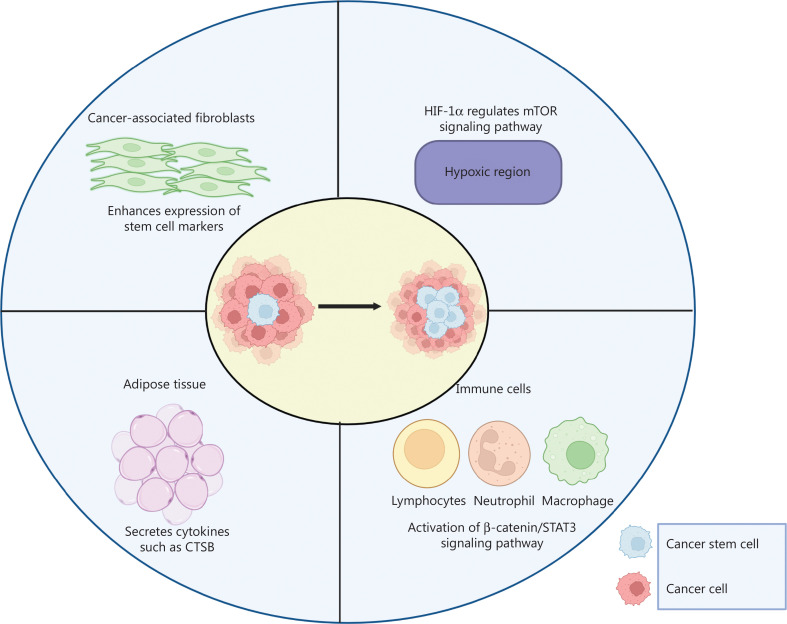
Roles of the tumor microenvironment in the regulation of cancer stem cells. The tumor microenvironment is selectively hypoxic and contains cancer-associated fibroblasts, immune cells, and adipose tissue. In the hypoxic region, HIF-1α regulates mTOR signaling. Cancer-associated fibroblasts enhance the expression of various stem cell markers. Adipose tissue secretes cytokines such as cathepsin B (CTSB). Immune cells activate various pathways, such as β-catenin and STAT3 signaling.

Genetic or epigenetic modifications within tumor cells as well as changes in the TME (including tumor cells, tumor stromal cells, and the non-cellular components of the extracellular matrix), facilitate tumor formation, maintenance, and progression^85^ (**Figure 4**). Tumor cells are considered the main TME component because they use various cellular, non-cellular, and non-malignant processes for their tumorigenesis during every stage of cancer development and metastasis^59,60^. Tumor cells can undergo EMT, wherein they lose their epithelial features, such as expression of E-cadherin and β-catenin, and acquire mesenchymal characteristics that increase the levels of N-cadherin and Vimentin^86^. EMT is associated with various events in the TME, such as tumor initiation, progression, cell migration, invasiveness, stemness, and resistance to therapy^87^. Recent findings suggest that, in prostate cancer, several common somatic mutations allow cancer cells to evade immunotherapy through modulation of the TME^88^. CSCs reside in their own “CSC niches” consisting of stromal cells, immune cells, growth factors, hypoxic areas, and extracellular matrix. The TME plays a critical role in maintaining the CSC population^89^. One important component of the TME is cancer-associated fibroblasts (CAFs), which regulate the tumorigenicity of various cancers, including prostate cancer. CAFs have been reported to enhance the gland-forming ability of PCSCs^90^. Furthermore, prostate carcinoma cells undergoing CAF-mediated EMT show elevated expression of CSC markers associated with the aggressiveness and metastasis of tumors. Consequently, treatments aimed at decreasing tumor growth and spread to secondary organs may be developed by limiting CAF-mediated EMT^91^.

Adipose tissues in the TME are involved in carcinogenesis, tumor progression, and metastasis. Moreover, adipocyte-secreted adipokines/cytokines play important roles in maintaining the CSC population^92^. Adipocytes co-cultured with prostate cancer cells secrete cysteine proteases, such as cathepsin B, and enhance the self-renewal property of CSCs^93^. The most abundant infiltrative immune cell population, tumor-associated macrophages (TAMs), account for 30%–50% of the tumor mass in the TME^94^. TAM-secreted CCL5 promotes the self-renewal ability of PCSCs, and prostate cancer invasion, EMT, and metastasis by stimulating the β-catenin/STAT3 pathway^95^. The rapid growth of cancer cells within the TME creates a hypoxic microenvironment. Therefore, the hypoxia-inducible factor 1α (HIF-1α) is activated in response to hypoxia in the TME. HIF-1α has been found to promote CSC populations in various cancers, including breast, bladder, and prostate cancer^96–98^. Marhold et al.^99^ have demonstrated that HIF-1α is involved in regulating mTOR signaling, survival, and metastasis of PCSCs. Hence, inhibition of this signaling pathway or HIF-1α may serve as potential therapeutic targets for PCSCs. CD133-positive PCSCs under hypoxic conditions stimulate HIF-1α gene expression, which is associated with an increase in N-Cadherin expression that leads to EMT and promotes tumor cell migration. Detailed mechanistic investigation of how regulation of CD133 in PCSCs leads to tumor progression is expected to provide a new therapeutic approach^100^.

## Metabolic reprogramming in prostate cancer and PCSCs

The major metabolic pathways are glycolysis, oxidative phosphorylation (OXPHOS), the tricarboxylic acid (TCA) cycle, glycogenolysis, lipogenesis, and the urea cycle. Cancer cells exhibit a transition in their metabolic needs; high demands for glucose must be met to enable survival, and additional nutrition is required^101,102^. This transition is called metabolic reprogramming. The metabolic reprogramming in heterogeneous cancer cells orchestrates genetic changes and/or epigenetic modifications^103^. The normal prostate gland synthesizes and secretes fluids that nourish and protect sperm by AR signaling-mediated processes. The AR is a nuclear hormone receptor transcription factor^104,105^. Acinar epithelial cells store zinc, thus inhibiting mitochondrial aconitase (ACO2), an enzyme catalyzing citrate oxidation, and blocking the entry of citrate into the TCA cycle^106^. Luminal cells secrete citrate as prostatic fluid to meet energy needs and support sperm viability through calcium chelation. In contrast, citrate is used in the TCA cycle primarily for energy production in other tissues^107^.

Metabolic reprogramming engages various metabolic pathways that support anabolic needs during cell proliferation and growth. The substantial shifts in nucleotide, amino acid, and lipid metabolism are due to genetic alterations in cancer cells. PCSCs exhibit low glycolysis, OXPHOS, and TCA, owing to their quiescent state^108^. Growth factors interact with tyrosine kinase receptors and activate the PI3K and AKT signaling pathways. This signaling cascade reprograms metabolism to support the anabolic requirements of cancer cells by reinforcing the activity of metabolic enzymes and nutrient transporters^109–111^. For example, genes such as c-Myc and KRAS often bear mutations affecting cellular metabolism in prostate cancer. Therefore, metabolic pathways (OXPHOS, amino acids, and lipid metabolism), AR signaling, neuroendocrine metabolism, Myc, and epigenetics drive metabolic reprogramming in prostate cancer and PCSCs.

## Prostate cancer and PCSC metabolism

### OXPHOS metabolism

Ample evidence has indicated that PCSCs, in contrast to non-PCSCs, use OXPHOS as an energy source. PCSCs preferentially use glucose to synthesize pyruvate, which is fed into the TCA cycle, whereas non-PCSCs show Warburg-like effects. Therefore, OXPHOS plays a crucial role in PCSC self-renewal and survival. PCSCs exhibit a metabolic switch to meet energy demands under physiological changes. PCSCs evade stress through chemoresistance, owing to their quiescent state. During glucose deprivation, PCSCs rely on OXPHOS to escape metabolic stress. Mass spectrometry has revealed high ACO2, fumarate hydrates, malate dehydrogenase-2, citrate synthase, and oxoglutarate dehydrogenase in prostate tumors^112^. A transcriptomic study performing metabolite analysis has shown higher levels of malate, succinate, and fumarate in prostate tumors than in adjacent non-cancerous tissue^113^. This finding suggests that the early stages of prostate cancer are more dependent on OXPHOS than aerobic glycolysis.

### Amino acid metabolism

Over the past 2 decades, many amino acids have been found to affect prostate cancer metabolism. AR signaling facilitates amino acid uptake by L-type amino acid transporters (LAT1 and LAT3), such as tryptophan, leucine, tyrosine, phenylalanine, and arginine^114^. Glutamine uptake and assimilation are coordinated by the overexpression of neutral amino acid transporters (ASCT1 and ASCT2). Glutamine is a crucial amino acid providing building blocks for TCA cycle metabolites and NADPH. The inhibition of these transporters prevents prostate cancer growth. These expression levels of these transporters could be helpful in diagnostic imaging to monitor prostate cancer progression in patients^114,115^. Androgen deprivation, an essential part of prostate cancer treatment, causes shrinkage of the normal prostate gland to 90% of its original size, owing to the loss of luminal cells^116^. Preclinical studies in LNCaP cells have shown an increase in ASCT2 expression in response to androgen treatment, thus enhancing the uptake of Fluciclovine, a leucine analog. Fluciclovine has been used in clinical trials to localize recurrent prostate cancer and prostatic metastases^117,118^. Ongoing AR-mediated amino acid metabolism research might provide new therapeutic approaches for the management of prostate cancer.

### Lipid metabolism

Lipids substantially contribute to the progression of prostate cancer^25^. Lipogenesis produces signaling molecules that serve as building blocks for lipid bilayers and cholesterol, thus enhancing intratumoral androgen synthesis^119–121^. The loss of PTEN in prostate cancer is associated with cholesterol accumulation in lipid droplets, thereby supporting tumor growth^122^. Moreover, PET agents, such as ^11^C acetate and ^11^C choline, involve lipid metabolism during prostate cancer metastasis. Expression of α-methyl acyl-CoA racemase (AMACR) is elevated and subsequently induces the fatty acid oxidation, an energy source in prostate cancer, independently of AR-mediated signaling^123–127^. Thus, a balance between lipid biosynthesis and fatty acid oxidation is critical for the survival and growth of prostate cancer. Dysregulation of the PI3K/AKT pathway leads to the overexpression of lipogenic enzymes and fatty acid synthetic enzymes in prostate cancer^128,129^. The ONCOMINE database has revealed differential expression of genes associated with lipid metabolism as well as fatty acid and cholesterol metabolism^130^. Cholesterol metabolism is essential for prostate cancer development. Circulating lipid droplets contain cholesterol esters that directly correlate with prostate cancer aggressiveness^122^. Therefore, inhibition of the cholesterol acetyltransferase enzyme (acetyl-CoA) has been used to block prostate cancer cell proliferation and invasion^131^. Loss of PTEN results in the hyperactivation of PI3K/AKT signaling, thereby activating lipid metabolism by upregulating SREBP-2 and low-density lipoprotein receptors^132^. The accumulation of cholesteryl esters is balanced by the action of transcription factors such as SREBP-2 and liver-X receptor (LXR). This accumulation is promoted by AR and AKT, which activate SREBP-2 and inactivate LXR. Statins, which are clinically used to regulate cholesterol, can also inhibit prostate cancer progression. Multiple reports have demonstrated the effect of statin therapy against prostate cancer mortality, owing to a decrease in PSA^133,134^. Further research on the role of statins in understanding the mechanism of prostate cancer progression may identify novel therapeutic targets for managing this cancer.

### AR-driven prostate cancer metabolism

The epithelial tissue of the prostate gland secretes testosterone, which is transformed into dihydrotestosterone (DHT) by 5α-reductase. The DHT then binds AR in the cytoplasm. The bound AR translocates into the nucleus and acts as a transcription factor regulating the expression of many genes, including KLK2/3 and NKX3-1^135^. Testosterone also promotes the synthesis of citrate and regulates the expression of zinc transporter (SLC39A1) and aspartate transporter (SLC1A1)^108,136^. The oncogenic metabolic reprogramming shifts to OXPHOS and the loss of zinc transporters in prostate tumors. The major zinc transporters encoded by SLC39 transporter sub-families are responsible for zinc absorption. Zinc depletion inhibits mitochondrial ACO2 and restores the TCA cycle. In malignant prostate cancer, the levels of citrate and zinc are lower than those in non-malignant cells. The hyperactive AR drives OXPHOS and lipogenesis, thereby promoting proliferation^137,138^. The AR also regulates the expression of glucose transporter (GLUT1), hexokinase 1/2 (HK1 and HK2), and glucose 6-phosphate dehydrogenase (G6PD), thereby regulating glycolysis, the pentose phosphate pathway, and lipogenesis^139,140^.

### NEPC metabolism

NEPC is an aggressive form of prostate cancer that develops because of selective pressure due to androgen removal. It is characterized by enhanced expression of neuroendocrine markers, such as neuron-specific enolase, chromogranin-A, and synaptophysin, along with decreased AR signaling^141^. Genetic loss of RB1 and TP53 and upregulation of MYCN and AURKA have been observed in NEPC. The loss of RN1 and TP53 further activates the pluripotency transcription factor SOX2 and epigenetic modifier EZH2^142,143^. The epigenetic changes are coupled to altered metabolism; for example, glycolysis produces pyruvate, which serves as a substrate for acetyl-CoA and consequently regulates histone acetyltransferase enzyme activity^144^. Histone lysine demethylase (KDM8) expression is upregulated, thus altering metabolism to favor aerobic glycolysis^145^. During treatment or disease progression, protein kinase C (PKC)λ is inhibited. Downregulation of PKCλ leads to increased serine biosynthesis *via* a mTORC1/ATF4-regulated pathway. The metabolic reprogramming increases cellular proliferation and S-adenosyl methionine, thereby inducing epigenetic changes that facilitate the further progression of NEPC^146^. NEPC is characterized by an increase in glycolysis and enhanced glutamine uptake, leading to increased pyruvate and acetyl-CoA production. Increased glycolysis and MCT-4-mediated lactic acid production and secretion are the most notable and clinically relevant metabolic characteristics in NEPC^12^.

### Myc-dependent reprogramming

Myc plays a crucial role in metabolic reprogramming because of its enhanced expression in prostate cancer and PCSCs. It regulates the glutamine transporter genes SLC1A4 and SLC1A5, thereby contributing to glutamine metabolism. It also regulates glucose metabolism *via* glucose transporter GLUT1, hexokinase 2 (HK2), enolase 1, lactate dehydrogenase A, and phosphofructokinase (PFK1)^147^. Therefore, Myc can be targeted to inhibit the growth of PCSCs^148,149^.

## Therapeutic strategies targeting PCSCs

Prostate cancer is a major malignancy in men, and prior studies have improved the understanding of the molecular basis of carcinogenesis, early diagnosis, and effective therapy. ADT is used primarily for prostate cancer treatment. Although ADT is effective, it targets only prostate cancer cells that are androgen-dependent; in most patients, the tumor then progresses to metastatic castration-resistant prostate cancer^150^. Furthermore, poor prognosis is often observed in such cases.

Patients with prostate cancer are treated with ADT in early disease stages^151^. Later, ADT and chemotherapy are given to patients with CRPCs to inhibit metastasis by targeting fast-growing prostate cancer cells^152,153^. The PCSCs show resistance to chemotherapy, hormone therapy, and radiotherapy. Therefore, prostate cancer may relapse because of the presence of undifferentiated CSCs. Current treatments usually destroy differentiated and rapidly dividing prostate cancer cells, thus leaving a resistant CSC subpopulation as a result of the heterogeneity of prostate cancer^154–156^. Various mechanisms primarily based on AR signaling may explain the establishment of CRPC. Targeting of AR signaling in prostate cancer cells has been the prime focus^44,157–159^. A recent report has shown that SOX2 plays a crucial role in the survival and pluripotency of PCSCs. It also promotes tumor aggressiveness. Moreover, SOX2-positive PCSCs have higher Gleason scores than SOX2-negative PCSCs. In addition, AR signaling suppresses SOX2 expression in CRPCs, and this effect can be reversed with anti-androgen factor exposure. SOX2 overexpression in castrated nude mice increases the tumor formation^160^. Thus, the relapse of prostate cancer to castration-resistant-SOX2 expressing tumors might be due to the constitutive expression of AR splice variant receptors that are deficient in the ligand binding domain^161–164^.

Another report has revealed that ADT increases the expression of AR and AR splicing variants and enriches the PCSC population^165^. Therefore, more advanced and effective therapy is required to target PCSCs. Metformin has been used as an anti-cancer therapy because it affects CSCs in various cancer types, including prostate cancer. It acts on mitochondria and decreases ATP synthesis *via* oxidative phosphorylation, an energy hub for PCSCs^166–170^. Therefore, Metformin enhances sensitivity toward PCSCs when applied in combination with existing therapies. It also increases drug efficacy and inhibits relapse. Bilen et al.^171^ have reported the treatment efficacy of Metformin alone or in combination with other drugs. This study has further proposed that Metformin and/or Zyflamend might target PCSCs and tumor niches and maintain a dormant tumor state.

Additionally, Iliopoulos et al.^168^ have highlighted the role of Metformin in combination with other drugs to prevent relapse by using prostate cancer xenograft models. Moreover, various studies have shown that phytochemicals or plant extracts can eliminate CSC populations in multiple cancers, including prostate cancer^172,173^. Among these natural compounds, curcumin has been found to be effective against prostate cancer. It affects cell proliferation through WNT signaling in AR-dependent and independent prostate cancer cell lines^174^. Furthermore, curcumin targets CD133^hi^/CD44^+/hi^ prostate cancer cells and decreases the PCSC population by inhibiting stemness-associated genes and preventing drug resistance^175^. Therefore, investigations have provided a deeper understanding of the association of PCSCs with tumorigenesis. PCSCs have also been reported to downregulate the expression of immunogenic markers such as HLA1 and PD1 while upregulating IL-4, thus providing a better understanding of the immune evasion abilities of undifferentiated cells^176^. Active immunotherapy by activating endogenous T cells to cancer *via* tumor-associated antigens can be performed with vaccines such as whole cell, peptide, or dendritic cell (DC) vaccines. Moreover, DCs, along with irradiated PCSCs, have a more targeted tumor response than DCs with irradiated non-CSCs. A DC-CSC-based vaccine has been found to inhibit mouse tumor growth^177^. Several clinical trials are in the pipeline to verify the effectiveness of DNA-based immunization in inducing antigen-specific T cells. In human CRPC, both prostatic acid phosphatase and PSA have been used as DNA-based vaccination targets in a randomized phase II trial^178,179^. Further studies are required to determine the key signaling regulators involved in the self-renewal and survival of PCSCs. The understanding gained should enable better therapeutic strategies to eliminate tumors, drug resistance, and relapse and increase patient survival.

## Conclusions

Mounting evidence in recent years indicates that CSCs are at the center of prostate cancer progression, metastasis, drug resistance, and, most importantly, relapse. In prostate cancer, disease recurrence leads to the development of CRPC. PCSCs can be isolated and enriched through multiple methods based on cell surface markers such as CD44, CD133, and α2β1 integrin; intracellular markers including Yamanaka factors and ABCG members; self-renewal; AR and PSA expression; and even cell size. Subsequent studies, including those performing isolation of PCSCs, have provided extensive insight into the regulatory mechanisms and signaling pathways involved in the induction and maintenance of CSCs. The various signaling pathways involved in PCSC induction and maintenance include the PI3K/AKT, RAS/MAPK, Hedgehog, Notch, WNT, and Hippo pathways. Through an intricate and extensive network of crosstalk between signaling molecules and various metabolic regulators, these pathways confer self-renewal ability, EMT potential, and drug resistance on prostate cancer cells.

The metabolic reprogramming in cancer cells, particularly in PCSCs, with reduced glycolysis, OXPHOS, and TCA, leads to the deregulation of downstream signaling, such as the PI3K/AKT axis. These pathways are associated with CSC maintenance. Thus, metabolic reprogramming and crosstalk between cellular signaling pathways can be concluded to promote stem-like characteristics, such as self-renewal, drug resistance, and EMT, in prostate cancer cells. This crosstalk between signaling and metabolic pathways provides molecular targets for developing novel therapeutics against PCSCs.

**Figure 5:**
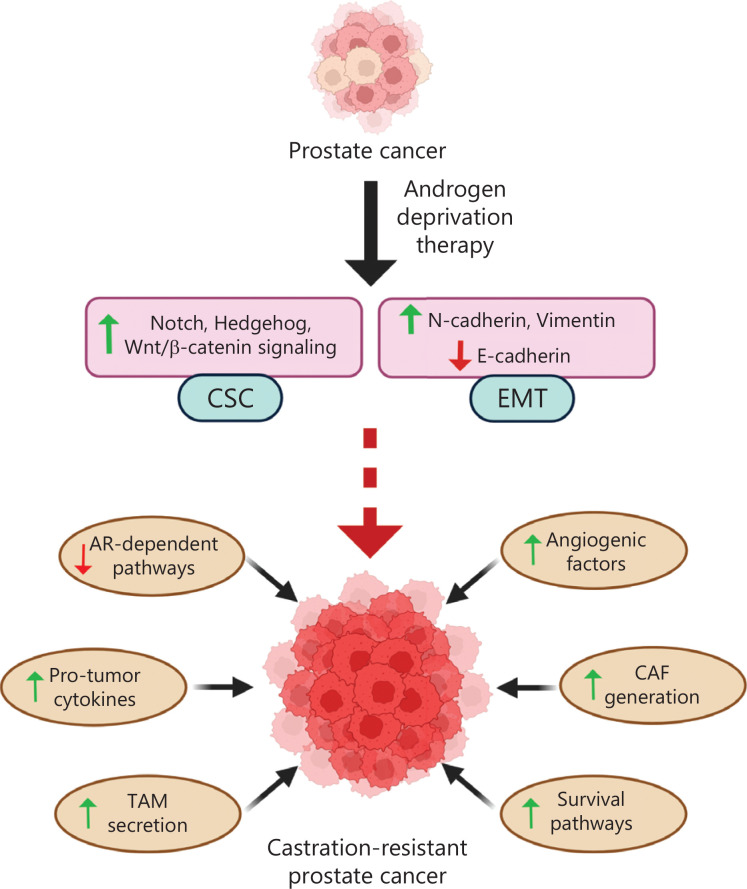
Androgen deprivation therapy leads to an aggressive prostate cancer type called castration-resistant prostate cancer (CRPC). Androgen deprivation therapy stimulates CSC pathways and the EMT process, thus leading to inhibition of androgen receptor (AR)-dependent pathways. However, alternative survival pathways are activated. Furthermore, angiogenic factor secretion, pro-tumorigenic cytokine secretion, TAMs, and CAFs also increase in the TME. These changes in the TME and targeted signaling pathways induce development of castration-resistant prostate cancer.

The presence of PCSCs, the EMT in prostate cancer cells, and CRPC are intrinsically connected. Emerging evidence indicates that EMT and the CSC phenomenon together contribute to the progression of prostate cancer in a hormone-independent manner. The ADT-mediated changes in the signaling pathways in CSCs, and in all cancer cells in general, lead to changes in the TME. The modified TME, in turn, regulates those signaling pathways within cancer cells (**Figure 5**). Reversing the EMT process or CSCs in prostate cancer could regress the development of CRPC. Thus, targeting the signaling crosstalk and reprogrammed metabolic pathways is a viable therapeutic approach for the better management of advanced prostate cancer.
